# A Comprehensive Analysis of *Triplophysa labiata* (Kessler, 1874) Mitogenome and Its Phylogenetic Implications within the *Triplophysa* Genus

**DOI:** 10.3390/genes14071356

**Published:** 2023-06-27

**Authors:** Chengxin Wang, Site Luo, Na Yao, Xinyue Wang, Yong Song, Shengao Chen

**Affiliations:** 1 College of Life Science and Technology, Tarim Research Center of Rare Fishes, Tarim University, CN-0997, Alar 843300, China120050013@taru.edu.cn (Y.S.); 2 School of Life Sciences, Xiamen University, Xiamen 361102, China; lstxmu@gmail.com

**Keywords:** *Triplophysa labiata*, mitochondrial genome, phylogeny analysis

## Abstract

In order to resolve the long-standing controversy surrounding the relationships within the *Triplophysa* genus, we conducted an extensive analysis of the complete mitogenome of *Triplophysa labiata* using DNBSEQ short reads. Additionally, we reconstructed the phylogeny of the Nemacheilidae family using mitogenome data. By comparing all available mitogenomes within the *Triplophysa* genus, we gained valuable insights into its evolutionary history. Our findings revealed that the mitogenome sequence of *T. labiata* is circular, spanning a length of 16,573 bp. It encompasses 13 protein-coding genes (PCGs), 22 transfer RNAs (tRNAs), 2 ribosomal RNAs (rRNAs), and a control region (D-loop). Among the PCGs, the start codon ATG was commonly observed, except in *cox1*, while the stop codons TAA/TAG/T were found in all PCGs. Furthermore, purifying selection was evident across all PCGs. Utilizing maximum likelihood (ML) methods, we employed the 13 PCGs and the concatenated nucleotide sequences of 30 *Triplophysa* mitogenomes to infer the phylogeny. Our results strongly supported the division of the *Triplophysa* genus into four primary clades. Notably, our study provides the first evidence of the close relationship between *T. labiata* and *T. dorsalis*. These findings serve as a significant foundation for future investigations into the mitogenomics and phylogeny of Nemacheilidae fishes, paving the way for further advancements in this field of research.

## 1. Introduction

Cypriniformes, the largest group of freshwater fish species globally, can be categorized into two primary superfamilies: Cyprinoidea and Cobitoidea. This classification encompasses a diverse array of aquatic organisms and reflects the extensive biodiversity within the Cypriniformes order [1]. The *Triplophysa* genus (Cobitoidea: Nemacheilidae), is an important and diverse group of fish species found in the Qinghai-Tibetan Plateau (QTP). The substantial uplift of the QTP over an extended duration is thought to have exerted a pivotal influence on the emergence and expansion of this genus. This geologically dynamic event is considered to be a key factor contributing to the evolutionary success and diversification of the studied genus [2,3]. The *Triplophysa* genus demonstrates exceptional adaptability to extreme environments, displaying remarkable temperature tolerance and resistance to desiccation. With its extensive distribution across the QTP, this genus offers an excellent opportunity to investigate the influence of historical climatic and geological events on present-day biodiversity. However, accurately identifying all species within this genus based solely on traditional taxonomy can be challenging due to their morphological variability, particularly among closely related species that share visual similarities [4,5,6,7,8,9,10,11,12,13,14,15,16,17,18,19,20,21,22,23,24].

**Figure 1:**
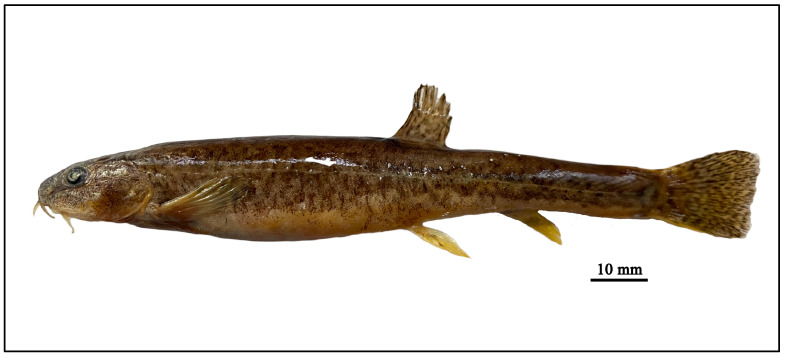
Photo. Of *T. labiata* by Shengao Chen.

Mitochondria are essential organelles found in nearly all eukaryotic organisms. They serve critical functions in regulating energy metabolism, apoptosis, aging, and various diseases. Consequently, mitochondria are widely recognized as indispensable components within the cells of most living organisms [3]. Double-stranded mitochondrial DNA (mtDNA) is a valuable molecular marker commonly utilized in systematic studies due to its simple structure, rapid evolutionary rate, abundant copies, and ease of isolation and purification. These characteristics make it a convenient and effective tool for investigating genetic relationships and phylogenetic patterns. As a result, it is widely regarded as an effective tool for investigating evolutionary relationships among species [25]. While more than 20 mitogenomes from the *Triplophysa* genus have been documented in the last 10 years, the mitogenome of *T. labiata* (Kessler, 1874) has not yet been reported [4,5,6,7,8,9,10,11,12,13,14,15,16,17,18,19,20,21,22,23,24]. *T. labiata* is a diminutive fish species that typically reaches a length of up to 10 cm when fully grown (Figure 1) [26]. *T. labiata* is a small cylindrical fish species with a rounded head and a diminutive mouth. It possesses a unique coloration that consists of a dark brown to blackish-brown body and a white to yellowish belly, with vertical dark bars that become less distinct towards the belly. The fins of this species are short and rounded, and its dorsal fin is located towards the rear end of the body. The native habitat of this species encompasses the high-altitude rivers and streams of the Tibetan Plateau and nearby regions. It exhibits a preference for the fast-flowing currents and rocky substrates found in the upper reaches of these water bodies. These habitats are characterized by cool temperatures, elevated oxygen levels, and clear, nutrient-poor water. Although the current conservation status of *T. labiata* according to the International Union for Conservation of Nature (IUCN) does not classify it as endangered, it faces potential threats from human-induced environmental degradation, including overfishing and pollution.

This study focuses on the comprehensive analysis of the mitochondrial genome (mitogenome) of *T. labiata*, a species belonging to the genus *Triplophysa*. By utilizing DNBSEQ short-read sequencing technology, we successfully assembled the complete mitogenome of *T. labiata*. The assembled mitogenomic data not only contributes to the understanding of *T. labiata*’s genetic makeup but also provides valuable insights into the phylogenetic relationships within the broader genus *Triplophysa*. Furthermore, this research expands the existing knowledge of the Nemacheilidae family by providing a robust mitogenomic dataset that can be utilized for future investigations into the phylogeny of Cypriniformes. The availability of complete mitogenomes enhances our ability to explore the evolutionary history and genetic diversity within this taxonomic group. The findings presented in this research article will lay the foundation for further studies on *Triplophysa* species and contribute to the broader field of fish phylogenetics.

## 2. Materials and Methods

### 2.1. Research Ethics Clearance

All procedures involving animal sampling and experimentation were performed in strict accordance with the guidelines and regulations for laboratory animal care. The Animal Care and Use Committee of Tarim University granted ethical approval for this study, with the assigned protocol code TDDKYXF20220316.

### 2.2. Experimental Fish and Sampling

A single adult *Triplophysa* labiata specimen was captured, using nets, from the Kunges River in the Xinjiang Uygur Autonomous Region of China, at coordinates 81°6′50.8″ E and 43°0′10.36″ N. The gender of the specimen was determined through examination of the dissected gonads. To ensure traceability and future reference, a voucher for the specimen was deposited at Tarim University under the accession number GYQ-S2022050002. Pectoral fin clips were carefully collected from the specimen and immediately preserved in 75% ethanol. Subsequently, the preserved fin clips were stored at a temperature of −80 °C until DNA isolation was performed. This rigorous sample preservation process ensures the integrity and quality of the genetic material, allowing for accurate and reliable analysis in subsequent molecular studies.

### 2.3. DNA Isolation, Library Preparation, and Sequencing

Genomic DNA extraction was performed using the TIANamp Genomic DNA Kit (TIANGEN, Beijing, China), a reliable and widely used method in molecular biology research. The extraction protocol provided by the manufacturer was followed meticulously to ensure high-quality DNA isolation from the collected *T. labiata* specimen. Subsequently, the extracted genomic DNA was utilized for DNBSEQ-T7 library preparation, a state-of-the-art sequencing library construction method known for its accuracy and efficiency. To achieve optimal library construction, 0.2 μg of the extracted genomic DNA was fragmented into pieces spanning approximately 350 bp in length. This fragmentation step enables the generation of short, overlapping DNA fragments that are suitable for subsequent sequencing processes [27]. 

The construction of the sequencing library was performed according to the manufacturer’s recommended guidelines to ensure accurate and reliable results. The library preparation process involved several key steps. Initially, the 5’ end of the library underwent phosphorylation and cyclization, which facilitates subsequent amplification and sequencing. Amplification was carried out using the rolling loop technique, enabling efficient and uniform replication of the library fragments. The resulting DNA nanospheres (DNBs) containing the amplified library were loaded onto the flow cell, and sequencing was conducted on the MGI DNBSEQ-T7 platform. This advanced sequencing platform is widely recognized for its high-throughput capabilities and exceptional sequencing accuracy. In our study, a total of 6 gigabases (Gb) of short reads were generated, providing substantial coverage of the *T. labiata* genome. To ensure the quality of the sequence data, we employed FastQC (v. 0.11.5), a popular quality control tool widely used by the genomics community. FastQC evaluates various quality metrics, including sequence quality scores, GC content, and sequence length distributions. Any low-quality reads were identified and subsequently filtered out using Fastp (v. 0.23), based on user-defined quality thresholds [28].

During the quality control process, reads that did not meet the predefined quality criteria were discarded to ensure the reliability of the sequencing data. Specifically, reads containing over 50% bases with a Q-value (quality score) lower than 2 were excluded from further analysis. Reads containing more than 5% unknown nucleotides were also removed. By implementing these quality filters, we aimed to eliminate sequences with low-confidence base calls and reduce potential errors that could affect downstream analyses. This stringent quality control step ensures that the remaining reads maintain a high level of accuracy and reliability, providing a solid foundation for subsequent bioinformatic analyses and interpretations of the *T. labiata* genome.

### 2.4. T. labiata Mitogenome Assembly and Annotation

The assembly of the mitogenome was accomplished using the GetOrganelle pipeline (v1.7.7.0), with default parameters, employing the ‘animal_mt’ database as the seed reads [29]. Following the assembly of the *T. labiata* mitogenome, short reads obtained from DNBSEQ-T7 were aligned to the assembly using BWA (v. 0.7.17). Subsequently, assembly polishing was performed using Pilon (v. 1.24) to improve the accuracy and quality of the assembly [30,31]. The annotation of the *T. labiata* mitogenome was performed using Mitoz v. 3.4, with the identification of the 13 protein-coding genes (PCGs) based on their homology to the reference mitogenome [31]. In addition, MITOS (http://mitos.bioinf.uni-leipzig.de/index.py, accessed on 1 March 2023) was utilized to identify a total of 22 tRNAs and 2 rRNAs. Finally, mitogenome maps were generated using OGDRAW [32] (https://chlorobox.mpimp-golm.mpg.de/OGDraw.html, accessed on 1 March 2023).

### 2.5. Sequence Analyses

The base composition, codon usage patterns, and relative synonymous codon usage (RSCU) were analyzed using Codon W, a widely used tool for codon analysis. This analysis provided insights into the nucleotide composition and the preferences of codons in the *T. labiata* mitogenome [33]. To assess patterns of nucleotide diversity (Pi) and non-synonymous (Ka) to synonymous rate (Ks) ratios across the 13 protein-coding genes (PCGs) in *Triplophysa*, we utilized DnaSP (v. 6.12.03), a software widely used for population genetic analysis. This allowed us to calculate and analyze the Pi and Ka/Ks ratios, providing valuable insights into the genetic variation and evolutionary dynamics within *Triplophysa* [34]. To estimate the sequence diversity of each protein-coding gene (PCG), sliding window analyses were performed using DnaSP (v. 6.12.03). The window length was set to ≤100 bp, with a step size of 25 bp. This allowed us to examine variations within the PCGs and identify regions of particular interest. Additionally, genetic distances were estimated using the Kimura-2 parameter (K2P) in MEGA (v. 7.0). This analysis provided insights into the genetic dissimilarity and evolutionary relationships among the sequences studied. By combining these approaches, we gained a comprehensive understanding of the sequence diversity and genetic distances within *Triplophysa* [35].

### 2.6. Phylogenetic Analyses

To elucidate the phylogenetic relationships between *T. labiata* and other species within the *Triplophysa* genus, the 13 protein-coding genes (PCGs) of *T. labiata* were concatenated with PCGs from other relevant species obtained from GenBank (Table 1). Multiple sequence alignment was performed using the default parameters in MAFFT. This alignment allowed us to compare the nucleotide sequences and identify conserved regions across the species.

To determine the phylogenetic relationships between *T. labiata* and other species in the *Triplophysa* genus, the 13 concatenated PCGs of *T. labiata* and other available species from GenBank (Table 1) were aligned using the default parameters in MAFFT [36]. To determine the optimal model for our phylogenetic analysis, we employed ModelFinder, which utilizes the Akaike information criterion (AIC). The AIC assesses the goodness of fit and the complexity of different models to identify the most suitable model for the given data. By comparing multiple models, we selected the model that best-balanced accuracy and simplicity based on the AIC score [37]. To construct a maximum-likelihood phylogenetic tree, we employed IQ-TREE (v. 2.1.2) with the GTR+F+R6 model. The robustness of the inferred tree topology was evaluated by performing 1000 ultrafast bootstraps. These bootstraps provided statistical support for the branches in the tree, allowing for an assessment of the reliability of the phylogenetic relationships [38].

**Table 1:** Detailed information on the mitogenome sequences belonging to 30 *Triplophysa* and 1 *Aborichthys* species retrieved from the NCBI database and analyzed in this study.

Genus	Species	Size (bp)	Accession No	Resource
*Triplophysa*	*T. labiata*	16,573	OQ559481	this study
	*Triplophysa bombifrons*	16,568	OP499856	[23]
	*Triplophysa bombifrons*	16,569	NC_027189	[20]
	*Triplophysa tenuis*	16,571	NC_030511	
	*Triplophysa dorsalis*	16,572	NC_029423	[16]
	*Triplophysa strauchii*	16,590	NC_026714	[20]
	*Triplophysa stoliczkai*	16,571	NC_017890	
	*Triplophysa xichangensis*	16,570	NC_030513	
	*Triplophysa dalaica*	16,569	NC_037925	
	*Triplophysa wuweiensis*	16,681	NC_030512	
	*Triplophysa venusta*	16,574	NC_029330	[19]
	*Triplophysa angeli*	16,569	NC_065113	
	*Triplophysa bleekeri*	16,568	NC_018774	[12]
	*Triplophysa anterodorsalis*	16,567	NC_024597	[11]
	*Triplophysa orientalis*	16,562	NC_030505	
	*Triplophysa stenura*	16,569	NC_032692	[13]
	*Triplophysa stewarti*	16,567	NC_030506	
	*Triplophysa lixianensis*	16,570	NC_030521	[8]
	*Triplophysa tibetana*	16,574	NC_030483	[10]
	*Triplophysa pappenheimi*	16,571	NC_037924	[7]
	*Triplophysa pappenheimi*	16,572	NC_033972	
	*Triplophysa robusta*	16,570	NC_025632	[22]
	*Triplophysa siluroides*	16,574	NC_024611	[6]
	*Triplophysa wangmoensis*	16,569	NC_037704	[9]
	*Triplophysa xiangxiensis*	16,598	NC_029492	[14]
	*Triplophysa rosa*	16,585	NC_019587	[15]
	*Triplophysa nasobarbatula*	16,605	NC_058005	[21]
	*Triplophysa baotianensis*	16,576	NC_056365	[5]
	*Triplophysa zhenfengensis*	16,564	NC_063617	[39]
	*Triplophysa yarkandensis*	16,574	NC_027517	[4]
*Aborichthys*	*Aborichthys elongatus*	16,544	NC_031582	

## 3. Results and Discussion

### 3.1. Genome Structure and Base Composition

**Figure 2:**
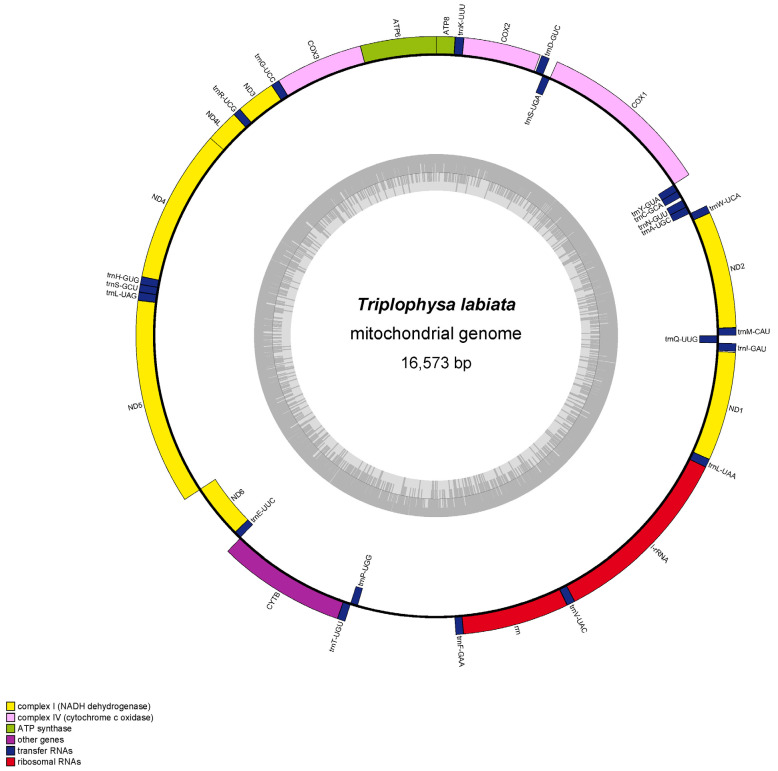
Circular representation of the *T. labiata* mitogenome is depicted, with the outer circle representing the H-strand and the inner circle representing the L-strand. The inner grey circle illustrates the distribution of GC and AT contents, with the dark and light regions denoting the GC and AT contents, respectively.

The circular double-stranded molecules of *T. labiata*’s complete mitogenome have been identified to be 16,573 bp in length. The mitogenome of *T. labiata* is characterized by a base composition of 28.14% A, 25.64% C, 18.81% G, and 28.41% T, with a slight bias towards AT nucleotides (56.55%). Consistent with other *Triplophysa* species, this mitogenome contains 13 protein-coding genes (PCGs), 22 transfer RNAs (tRNAs), 2 ribosomal RNAs (rRNAs), and a putative control region with a high AT content (Figure 2, Table 2). The lengths of the identified tRNAs in *T. labiata*’s mitogenome ranged from 66 bp to 76 bp, with tRNA^Cys^ being the shortest (66 bp) and tRNA^Lys^ being the longest (76 bp). Notably, the control region of *T. labiata*’s mitogenome spans 918 bp and is positioned between tRNA^Pro^ and tRNA^Phe^. The mitogenome of *T. labiata* exhibited a high degree of similarity with other *Triplophysa* species, with variations observed in specific mitochondrial gene regions (Table 1). Upon closer examination, differences were found to range from 29 bp to 308 bp. These variations were primarily identified in genes associated with the control region, highlighting potential genetic divergence and evolutionary patterns within the *Triplophysa* genus.

### 3.2. Description of Protein-Coding Genes (PCGs)

The *T. labiata*’s mitogenome exhibits a notable gene repertoire, with a total of 28 genes encoded by the H-strand, including *atp6, apt8, cox1, cox2, cox3, cob, nad1, nad2, nd3, nad4, nd4l*, *nad5*, *l-rRNA*, *s-rRNA, trnD*, *trnF*, *trnG*, *trnH*, *trnI*, *trnK*, *trnL*, *trnM*, *trnR*, *trnS*, *trnT*, *trnV*, and *trnW*. In contrast, the L-strand encodes nine genes, namely *nad6, trnQ, trnA, trnN*, *trnC*, *trnY*, *trnS*, *trnE*, *and trnP*. It is noteworthy that the gene order and orientation observed in this study closely resemble those reported in previous investigations of *Triplophysa* mitogenomes. Most protein-coding gene sequences start with the ATG start codon, except for *cox1*, which begins with GTG. Among the protein-coding genes, six terminate with the codon TAA (*cox1, atp8, apt6, nad4l, nad6, and nad1*), three conclude with TAG (*nad3, nad4, and nad5*), while the remaining four end with T (*nad2, cox2, cox3, and cob*). The arrangement and composition of genes in this study align with previous findings from mitogenome studies within the *Triplophysa* genus [11,40]. Considering the composition and arrangement of the *Triplophysa* mitogenome genes observed in this study, it is evident that the mitogenomic organization is highly conserved within the *Triplophysa* genus. Given the similarity in gene order and orientation observed in this study and previous investigations of *Triplophysa* mitogenomes, it can be inferred that these genes have the potential to contribute to phylogenetic resolution at the *Triplophysa* genus level. The conservation of gene composition and arrangement suggests a shared evolutionary history and can serve as a valuable resource for future phylogenetic studies in *Triplophysa*.

**Figure 3:**
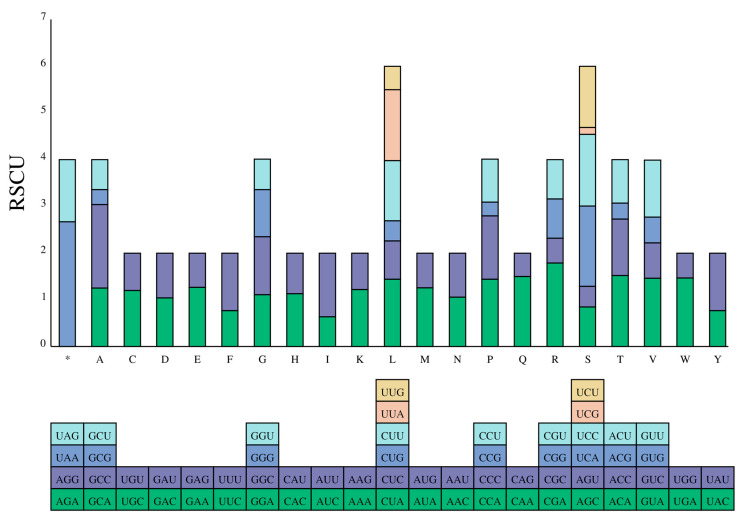
Bar plot showing the distribution of codon content of the amino acids in the 13 protein-coding genes (PCGs) of the *T. labiata* mitogenome. Note: “*” represents stop codon.

**Figure 4:**
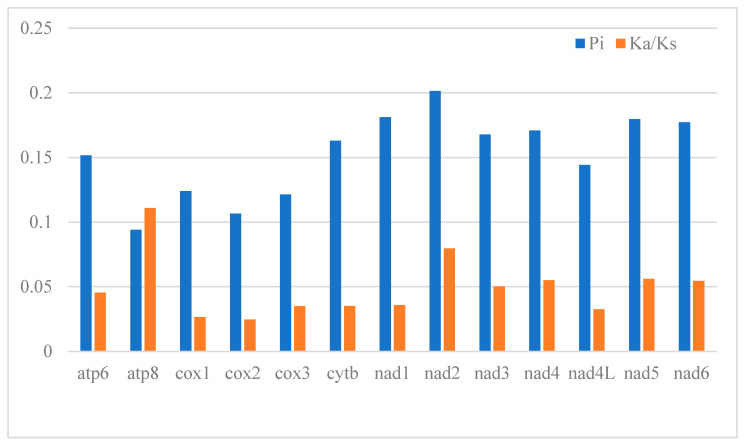
Genetic diversity (Pi) and the Ka/Ks ratio of each PCG in the *Triplophysa* mitogenome.

Codon preference is a significant factor influencing gene expression levels and mRNA stability, thereby offering valuable insights into evolutionary patterns and phylogenetic relationships [40]. The mitogenome of *T. labiata* contains 13 protein-coding genes, totaling 3809 codons (Figure 3). Notably, certain amino acids exhibited higher usage rates within these codons, with Isoleucine, Alanine, Leucine, Phenylalanine, Threonine, Methionine, and Valine being the most prevalent at 5.01%, 4.12%, 4.12%, 3.68%, 2.94%, 2.76%, and 2.36%, respectively (Table S1). Conversely, Serine had the lowest occurrence, comprising a mere 0.16% of all codons. An intriguing finding of this study was that the stop codon TAA was the most frequently utilized among the protein-coding genes in the *T. labiata* mitogenome. These findings underscore the significance of codon usage in unraveling evolutionary patterns and understanding phylogenetic relationships. The Ka/Ks ratio (ω) is a fundamental parameter utilized in molecular evolution phylogenetic analyses to identify molecular adaptation [40]. In our investigation, all 13 protein-coding genes exhibited Ka/Ks values below 1, with values ranging from <0.12 (Figure 4 and Table S2). These findings suggest that purifying selection exerted a substantial influence on these genes, rendering them suitable for elucidating the phylogenetic relationships within the *Triplophysa* genus. Notably, *atp8, nad2, nad5*, and *nad4* genes exhibited higher Ka/Ks ratios (0.111, 0.079, 0.056, and 0.055, respectively) compared to other protein-coding genes, indicating a more relaxed evolutionary pressure and greater retention of non-synonymous mutations. Interestingly, the *cox2* gene displayed the lowest Ka/Ks ratio, suggesting it experienced the most pronounced evolutionary pressure. Given that mitochondrial DNA encodes crucial components of the respiratory chain, it plays a critical role in mitochondria inheritance, rendering the mitogenome susceptible to the accumulation of deleterious mutations. The strong purifying selection acting on the *cox2* gene helps to eliminate deleterious mutations. This makes the *cox2* gene an ideal molecular marker for phylogenetic analysis within the *Triplophysa* genus. Based on these findings, it is reasonable to infer that these genes can contribute to phylogenetic resolution primarily at the genus level within the *Triplophysa* genus, with the potential to provide insights into the evolutionary relationships and divergence patterns within this group.

In genetics, nucleotide diversity is commonly denoted as π. It refers to the average differences or variability between two randomly selected nucleotide sequences within a given gene or genomic region. It measures the extent of variation or genetic diversity in a gene or genome and can be used to assess genetic diversity. A higher π value indicates a higher level of variation in nucleotide sequences for that particular region. To identify DNA polymorphisms, we analyzed the aligned sequences of 6 *Triplophysa* mitogenomes’ 13 protein-coding genes (Table S2). Analysis revealed that the highest nucleotide diversity (Pi) was observed in the nad2 gene (0.201), followed by nad1 (0.181), nad5 (0.179), and nad6 (0.177). Conversely, cox2 (0.106) and atp8 (0.094) exhibited the lowest values. Similar trends were observed while analyzing mean genetic distances (refer to Table S3). Specifically, high genetic distances were observed in the nad2, nad11, nad5, and nad6 genes, with values of 0.24, 0.21, 0.21, and 0.21, respectively. In contrast, the cox3, cox2, and atp8 genes exhibited lower genetic distances of 0.14, 0.12, and 0.10, respectively.

### 3.3. Phylogenetic Analyses

**Figure 5:**
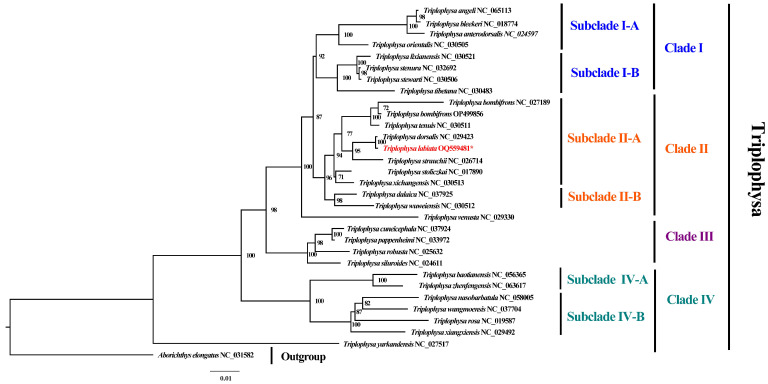
Phylogenetic relationships between 30 *Triplophysa* mitogenomes inferred by ML analyses, based on 13 PCGs. The number (%) on the branches indicates the maximum likelihood (ML) bootstrap support value (only values above 70% are shown on the tree), indicating the lower limit for the support of the tree branches. The *T. labiata* in this study is marked in red and an asterisk (*).

To ensure the robustness of our phylogenetic analyses, we retrieved all 29 *Triplophysa* mitogenomes available as of 17 April 2023 from the NCBI reference sequence (RefSeq) database. Based on our maximum likelihood (ML) analyses, we observed that *Triplophysa* can be classified into four main clades (Clades I, II, III, and IV) (Figure 5). Clade I further divided into two subclades (I-A and I-B), which are strongly supported by our phylogenetic reconstructions. Clade II comprises two subclades containing a total of 10 species, with *T. labiata* being the closest species to T. dorsalis in subclade II-A [18]. Subclade II-B includes *T. dalaica* and *T. wuweiensis*. Clade III consists of four species (*T. cuneicephala, T. pappenheimi, T. robusta*, and *T. siluroides*), while the remaining six species are assigned to Clade IV, which can be further divided into two subclades (Subclade IV-A and Subclade IV-B). Notably, *T. yarkandensis* occupies a basal position in the phylogenetic tree, suggesting that it may represent an ancestral group. Compared to other data sets, our analysis based on mitogenomes consistently reveals systematic relationships that align with the reported findings on *T. Bombifrons*. These results emphasize the clear division into distinct clades and subclades. These findings significantly enhance our understanding of the evolutionary history of *Triplophysa* and underscore the importance of conducting further investigations using complementary data sets to validate and expand upon these valuable observations.

## 4. Conclusions

In this study, we employed DNBSEQ short-read sequencing technology to present the first complete mitogenome sequence of *T. labiata*. Our findings demonstrate that the structure of the *T. labiata* mitogenome is consistent with that of the *Triplophysa* genus, comprising 13 protein-coding genes (PCGs), 22 tRNAs, 2 rRNAs, and a control region. Phylogenetic analysis based on the 13 PCGs strongly supports the classification of the *Triplophysa* genus into 4 main clades and reveals a close relationship between *T. labiata* and T. dorsalis. These results not only contribute to the expanding collection of mitogenomes in the *Triplophysa* genus but also enhance our understanding of the molecular characteristics of the Nemacheilidae family. Moreover, they establish a solid groundwork for future investigations into population genetics and phylogenetic relationships within this family. The analysis of purifying selection and Ka/Ks ratios in the protein-coding genes provides valuable insights into the evolutionary dynamics of *Triplophysa* species. These genes, which have experienced varying evolutionary pressures, hold promise as potential markers for population-level studies. By utilizing these markers, researchers can investigate important aspects of population genetics, including population differentiation, gene flow, and adaptive genetic variations. The identification of genes under different evolutionary pressures opens up avenues for assessing population-level phenomena. Researchers can calculate genetic diversity indices, examine the demographic history, and explore the impact of environmental factors on population structure. These population-level analyses contribute to a deeper understanding of the intricate dynamics within *Triplophysa* populations.

In summary, our genus-level study lays a solid foundation for expanding research into investigations of the *Triplophysa* genus. The identified genetic markers, in combination with the expanded genetic data set and established systematic relationships, provide valuable tools and resources for exploring population dynamics, genetic diversity, and adaptation within the *Triplophysa* genus. This research contributes to the broader field of population genetics and facilitates a more comprehensive understanding of the evolutionary processes occurring within *Triplophysa*.

## Figures and Tables

**Table 2:** Annotation of genes in the *T. labiata* mitogenome.

Locus	Start	Stop	Size (bp)	Start Coding	Stop Coding	Strand
*tRNA*Met	1	69	69			H
*nad2*	70	1116	1047	ATG	T	H
*tRNA*Trp	1115	1184	70			H
*tRNA*Ala	1187	1255	69			L
*tRNA*Asn	1257	1329	73			L
*tRNA*Cys	1361	1426	66			L
*tRNA*Tyr	1427	1495	69			L
*cox1*	1497	3047	1551	GTG	TAA	H
*tRNA*Ser	3048	3118	71			L
*tRNA*Asp	3121	3193	73			H
*cox2*	3207	3911	705	ATG	T	H
*tRNA*Lys	3898	3973	76			H
*atp8*	3975	4142	168	ATG	TAA	H
*atp6*	4133	4816	684	ATG	TAA	H
*cox3*	4816	5616	801	ATG	T	H
*tRNA*Gly	5600	5672	73			H
*nad3*	5673	6023	351	ATG	TAG	H
*tRNA*Arg	6022	6091	70			H
*nad4l*	6092	6388	297	ATG	TAA	H
*nad4*	6382	7764	1383	ATG	TAG	H
*tRNA*His	7764	7833	70			H
*tRNA*Ser	7834	7901	68			H
*tRNA*Leu	7903	7975	73			H
*nad5*	7976	9814	1839	ATG	TAG	H
*nad6*	9811	10,332	522	ATG	TAA	L
*tRNA*Glu	10,333	10,401	69			L
*cob*	10,406	11,566	1161	ATG	T	H
*tRNA*Thr	11,547	11,617	71			H
*tRNA*Pro	11,616	11,685	70			L
*tRNA*Phe	12,604	12,672	69			H
*12S rRNA*	12,673	13,622	950			H
*tRNA*Val	13,625	13,696	72			H
*16S rRNA*	13,697	15,374	1678			H
*tRNA*Leu	15,375	15,449	75			H
*nad1*	15,450	16,424	975	ATG	TAA	H
*tRNA*Ile	16,432	16,503	72			H
*tRNA*Gln	16,502	16,572	71			L

## Data Availability

The genome sequence data that support the findings of this study are openly available in the GenBank of NCBI at (https://www.ncbi.nlm.nih.gov/) under accession no OQ559481 (accessed on 5 April 2023). The associated BioProject, SRA, and Bio-Sample numbers are PRJNA956153, SAMN34205561, and SRR24183507, respectively.

## Supplementary Materials

The following supporting information can be downloaded at: https://www.mdpi.com/article/10.3390/genes14071356/s1. Table S1: Amino acid composition and relative synonymous codon usage in the mitogenomes of *T. labiata*, Table S2: Nucleotide diversity, Ka, and Ks results of *Triplophysa,* Table S3: Genetic distances, based on 13 PCGs of *Triplophysa*.

## References

[1] Nelson J.S., Grande T.C., Wilson M.V. (2016). Fishes of the World.

[2] Wen J., Yu Y., Xie D.-F., Peng C., Liu Q., Zhou S.-D., He X.-J. (2020). A transcriptome-based study on the phylogeny and evolution of the taxonomically controversial subfamily Apioideae (Apiaceae). Ann. Bot..

[3] He B., Zhao Y., Su C., Lin G., Wang Y., Li L., Ma J., Yang Q., Hao J. (2023). Phylogenomics reveal extensive phylogenetic discordance due to incomplete lineage sorting following the rapid radiation of alpine butterflies (Papilionidae: *Parnassius*). Syst. Entomol..

[4] Ning X., Zhang Y.-Z., Sui Z.-H., Quan X.-Q., Zhang H.-G., Liu L.-X., Han Q.-D., Liu Y.-G. (2020). The complete mitochondrial DNA sequence of Kashgarian loach (*Triplophysa yarkandensis*) from Bosten Lake. Mitochondrial DNA Part B.

[5] Wang Y., Xiao N., Wang S., Luo T., Yang X., Liu T., Zhou J. (2021). The complete mitochondrial genome of a cave-dwelling loach *Triplophysa baotianensis* (Teleostei: Nemacheilidae). Mitochondrial DNA Part B.

[6] Chen I.-S., Liu G.-D., Prokofiev A.M. (2016). The complete mitochondrial genome of giant stone loach *Triplophysa siluroides* (Cypriniformes: Balitoridae). Mitochondrial DNA Part A.

[7] Feng X., Chen Y., Sui X., Chen Y. (2019). The complete mitochondrial genome of *Triplophysa cuneicephala* (Cypriniformes: Balitoridae) with phylogenetic consideration. Mitochondrial DNA Part B.

[8] Jing H., Yan P., Li W., Li X., Song Z. (2016). The complete mitochondrial genome of *Triplophysa lixianensis* (Teleostei: Cypriniformes: Balitoridae) with phylogenetic consideration. Biochem. Syst. Ecol..

[9] Liu T., You P. (2016). The complete mitochondrial genome of *Triplophysa* sp. (Teleostei: Cypriniformes: Balitoridae). Mitochondrial DNA Part A.

[10] Wang J., Li L., Jin X., Wang P., Du Y., Ma B. (2019). The complete mitochondrial genome of *Triplophysa tibetana*. Mitochondrial DNA Part B..

[11] Que Y., Liao X., Xu D., Yang Z., Tang H., Zhu B. (2016). The complete mitochondrial genome sequence of *Triplophysa anterodorsalis* (Teleostei, Balitoridae, Nemacheilinae). Mitochondrial DNA Part A.

[12] Tang Q., Huang Y., Wang J., Huang J., Wang Z., Peng Z. (2013). The complete mitochondrial genome sequence of *Triplophysa bleekeri* (Teleostei, Balitoridae, Nemacheilinae). Mitochondrial DNA.

[13] Yan Y., Luo D. (2016). The complete mitochondrial genome sequence of *Triplophysa stenura* (Teleostei, Cypriniformes): Genome characterization and phylogenetic analysis. Mitochondrial DNA Part B.

[14] Wang X., Cao L., Zhang E. (2017). The complete mitochondrial genome sequence of *Triplophysa xiangxiensis* (Teleostei: Nemacheilidae). Mitochondrial DNA Part A.

[15] Wang J., Tang Q., Wang Z., Zhang Y., Wu Q., Peng Z. (2012). The complete mitogenome sequence of a cave loach *Triplophysa rosa* (Teleostei, Balitoridae, Nemacheilinae). Mitochondrial DNA.

[16] Lei D., Conteh Kanu U., Zhao G., Xie P., Yuan H., Li Y., Niu J., Ma X. (2016). The complete mtDNA genome of *Triplophysa dorsalis* (Cypriniformes, Balitoridae, Cobitoidea): Genome characterization and phylogenetic analysis. Mitochondrial DNA Part A.

[17] Sun X., Cheng J. (2022). Comparative mitogenomic analyses and new insights into the phylogeny of thamnocephalidae (branchiopoda: Anostraca). Genes.

[18] Greaves L.C., Elson J.L., Nooteboom M., Grady J.P., Taylor G.A., Taylor R.W., Mathers J.C., Kirkwood T.B.L., Turnbull D.M. (2012). Comparison of Mitochondrial Mutation Spectra in Ageing Human Colonic Epithelium and Disease: Absence of Evidence for Purifying Selection in Somatic Mitochondrial DNA Point Mutations. PLoS Genet..

[19] Wang C., Liang Y.-Q., Li M., Zhang Y., Shen Z.-J., Jiang Z.-W. (2016). Complete mitochondrial DNA genome of *Triplophysa venusta* (cypriniformes: Cobitida). Mitochondrial DNA Part A.

[20] Ming Han M., Lu J., Wang L., Mahboob S., Al-Ghanim K.A., Sun X.-W. (2016). Complete mitochondrial genome of the *Triplophysa bombifrons* and *Triplophysa strauchii*. Mitochondrial DNA Part A.

[21] Yang X., Wen H., Luo T., Zhou J. (2020). Complete mitochondrial genome of *Triplophysa nasobarbatula*. Mitochondrial DNA Part B.

[22] Yan P., Li J., Ma Q., Deng Y., Song Z. (2016). Complete mitochondrial genome of *Triplophysa robusta* (teleostei: Cypriniformes: Balitoridae). Mitochondrial DNA Part A.

[23] Wang X., Song Y., Xie H., Zi F., Chen S., Luo S. (2023). Complete mitogenome of the *Triplophysa bombifrons*: Comparative analysis and phylogenetic relationships among the members of *Triplophysa*. Genes.

[24] Kim N.Y., Ahn S.J., Seo J.S., Jeon E.J., Cho M.Y., Choi H.S. (2022). Characterization of the complete mitochondrial genome of *Miamiensis avidus* causing flatfish scuticociliatosis. Genetica.

[25] Boore J.L. (1999). Animal mitochondrial genomes. Nucleic Acids Res..

[26] Cao L., Causse R., Zhang E. (2012). Revision of the loach species *Barbatula nuda* (Bleeker 1865) (Pisces: Balitoridae) from North China, with a description of a new species from Inner Mongolia. Zootaxa.

[27] Jeon S.A., Park J.L., Park S.-J., Kim J.H., Goh S.-H., Han J.-Y., Kim S.-Y. (2021). Comparison between MGI and Illumina sequencing platforms for whole genome sequencing. Genes Genom..

[28] Chen S., Zhou Y., Chen Y., Gu J. (2018). fastp: An ultra-fast all-in-one FASTQ preprocessor. Bioinformatics.

[29] Jin J.-J., Yu W.-B., Yang J.-B., Song Y., Yi T.-S., Li D.-Z. (2018). GetOrganelle: A simple and fast pipeline for de novo assembly of a complete circular chloroplast genome using genome skimming data. BioRxiv.

[30] Li H. (2013). Aligning Sequence Reads, Clone Sequences and Assembly Contigs with BWA-MEM. arXiv.

[31] Walker B.J., Abeel T., Shea T., Priest M., Abouelliel A., Sakthikumar S., Cuomo C.A., Zeng Q., Wortman J., Young S.K. (2014). Pilon: An Integrated Tool for Comprehensive Microbial Variant Detection and Genome Assembly Improvement. PLoS ONE.

[32] Greiner S., Lehwark P., Bock R. (2019). OrganellarGenomeDRAW (OGDRAW) version 1.3.1: Expanded toolkit for the graphical visualization of organellar genomes. Nucleic Acids Res..

[33] Iriarte A., Lamolle G., Musto H. (2021). Codon usage bias: An endless tale. J. Mol. Evol..

[34] Rozas J., Ferrer-Mata A., Sánchez-DelBarrio J.C., Guirao-Rico S., Librado P., Ramos-Onsins S.E., Sánchez-Gracia A. (2017). DnaSP 6: DNA sequence polymorphism analysis of large data sets. Mol. Biol. Evol..

[35] Horiike T. (2016). An introduction to molecular phylogenetic analysis. Rev. Agric. Sci..

[36] Katoh K., Standley D.M. (2013). MAFFT multiple sequence alignment software version 7: Improvements in performance and usability. Mol. Biol. Evol..

[37] Kalyaanamoorthy S., Minh B.Q., Wong T.K.F., von Haeseler A., Jermiin L.S. (2017). ModelFinder: Fast model selection for accurate phylogenetic estimates. Nat. Methods.

[38] Nguyen L.-T., Schmidt H.A., von Haeseler A., Minh B.Q. (2015). IQ-TREE: A Fast and Effective Stochastic Algorithm for Estimating Maximum-Likelihood Phylogenies. Mol. Biol. Evol..

[39] Carraretto D., Aketarawong N., Di Cosimo A., Manni M., Scolari F., Valerio F., Malacrida A.R., Gomulski L.M., Gasperi G. (2020). Transcribed sex-specific markers on the Y chromosome of the oriental fruit fly, *Bactrocera dorsalis*. BMC Genet..

[40] Zhou L., Huang S., Wang Q., Li Z., Li Z., He A., Chen J., Liu L., Zou K. (2022). Novel evolutionary insights into nemacheilid cavefish: Evidence from comparative analysis of mitochondrial genomes. J. Oceanol. Limnol..

